# Customers’ Perceptions of Compliance with a Tobacco Control Law in Restaurants in Hanoi, Vietnam: A Cross-Sectional Study

**DOI:** 10.3390/ijerph15071451

**Published:** 2018-07-10

**Authors:** Anh Kim Dang, Bach Xuan Tran, Long Hoang Nguyen, Hoa Thi Do, Cuong Tat Nguyen, Mercedes Fleming, Huong Thi Le, Quynh Ngoc Hoang Le, Carl A. Latkin, Melvyn W. B. Zhang, Roger C. M. Ho

**Affiliations:** 1 Institute for Preventive Medicine and Public Health, Hanoi Medical University, Hanoi 100000, Vietnam; bach.ipmph@gmail.com (B.X.T.); lethihuong@hmu.edu.vn (H.T.L.); 2 Bloomberg School of Public Health, Johns Hopkins University, Baltimore, MD 21205, USA; carl.latkin@jhu.edu; 3 Department of Public Health Sciences, Karolinska Institutet, SE-171 77 Stockholm, Sweden; longnh.ph@gmail.com; 4 Department of Nutrition and Food Safety, Hanoi Medical University, Hanoi 100000, Vietnam; dothihoa1954@yahoo.com; 5 Institute for Global Health Innovations, Duy Tan University, Da Nang 550000, Vietnam; cuong.ighi@gmail.com; 6 School of Medicine and Medical Science, University College Dublin, Dublin 4, Ireland; mercyfleming@gmail.com; 7 Faculty of Pharmacy, Duy Tan University, Duy Tan 550000, Vietnam; quynhle2k01@gmail.com; 8 Biomedical Global Institute of Healthcare Research & Technology (BIGHEART), National University of Singapore, Singapore 117599, Singapore; ciezwm@nus.edu.sg; 9 Department of Psychological Medicine, Yong Loo Lin School of Medicine, National University of Singapore, Singapore 119228, Singapore; pcmrhcm@nus.edu.sg

**Keywords:** smoking, secondhand smoke, tobacco, restaurant, law, Vietnam

## Abstract

The Tobacco Harm Prevention Law has been promulgated in 2012 in Vietnam, prohibiting smoking in public places such as restaurants except for designated smoking areas. However, currently, evidence about Vietnamese customers’ and restaurants’ compliance with the Law is constrained. This study aimed to explore customers’ perceptions; attitudes and practices towards the compliance with tobacco control regulations in the restaurants in Hanoi, Vietnam. A cross-sectional study was performed in October 2015 with 1746 customers in 176 communes in Hanoi, Vietnam. Data about customers’ perceptions on how restaurants comply with the smoking control law and whether customers smoking actively or experienced SHS in restaurants in the last 30 days were collected. Multivariable mixed effects logistic regression model was used to determine the factors related to smoking in the restaurant. Most customers were aware of the law on Tobacco Harm Prevention (79%; *n* = 1320) and regulations that prohibited smoking in restaurants (78.4%; *n* = 1137). While 75.8% (*n* = 1285) of customers perceived that they did not see or rarely saw no-smoking signs, 17.7% (*n* = 481) of customers reported that they frequently saw direct marketing of tobacco in visited restaurants. About one-fourth of customers witnessed that the staff reminded customers not to smoke inside restaurants (28.8%; *n* = 313), and 65% (*n* = 1135) sometimes or always were exposed to secondhand smoke in their visited restaurants. People who were female (OR = 0.02, 95% CI = 0.01–0.05) were less likely to report their smoking in the restaurant than their counterparts. Those having higher age (OR = 1.03; 95% CI = 1.01–1.06), high school education (OR = 2.14, 95% CI = 1.07–4.26), being office workers (OR = 3.24, 95% CI = 1.33–7.92) or unemployed (OR = 4.45; 95% CI = 1.09–18.15) had a higher likelihood of reporting to be restaurant smokers than those having lower high education or students, respectively. This study highlighted a low level of perceived compliance with the smoke-free law in Vietnamese restaurants. Improving the monitoring systems for the enforcement of the smoking law in restaurants should be prioritized; restaurant owners should implement 100% smoke-free environments as following the best practice towards the tobacco control law along with educational campaigns to promote the awareness of restaurant owners and customers about the tobacco control law.

## 1. Introduction

Tobacco smoking has been well-documented as a common issue in restaurants [1,2,3,4,5]. Given a growing concern to protect employees and patrons from the adverse health effects of smoking exposure, banning smoking in restaurants has been prioritized in global tobacco control ordinances [6]. At an international level, the World Health Organization (WHO) called for the need to protect non-smokers and allow them to live in a smoke-free environment as per Article 8 of the Framework Convention on Tobacco Control (FCTC) [7]. The WHO also developed an MPOWER comprehensive strategy based on the FCTC in 2008, including six tobacco control measures that promote mandates on smoke-free public places [8]. At the national level, many countries such as Australia, Canada, the United Kingdom, Singapore, Malaysia, Thailand and others have adopted the tobacco control legislation to restrict smoking in food establishments [9,10,11,12,13,14]. Previous studies found that these regulations promoted a significant reduction of secondhand smoke [15,16], increased motivation to quit smoking [17] and, in particular, did not cause any loss of business profits [18,19,20,21].

Despite existing efforts, the compliance with smoke-free policies varies among nations depending on social acceptability and attitudes related to smoking behaviors [17,22,23]. Compliance rates are higher in developed countries namely the United States (95.8%), Australia (98%), Canada (94.5%), the United Kingdom (79.6%) [24], and China (85.2%) [25]. Meanwhile, low compliance rates are reported in developing countries such as South Africa (1.8%) [26], Guatemala (29%) [27], Malaysia (40%), and Thailand (57%) [10].

In Vietnam, passive smoking exposure in the restaurant is a public health crisis. According to the Global Adult Tobacco Survey (GATS) in 2010, 23.8% of the Vietnamese population (47.4% of men and 1.4% of women) currently smoke tobacco, and the rate of passive smoking in restaurants was considerably high (84.9%) [2]. To counter this epidemic, the Government of Vietnam ratified the WHO FCTC in 2009 and mandated smoking restrictions in indoor public places in 2010 [28]. In 2012, the comprehensive Law on Prevention and Control of Tobacco Harms (Law No 09/2012/QH13) was enacted by the National Assembly in order to enforce stricter regulations in controlling tobacco smoke exposure in public places [29]. The policy aims to prohibit smoking in indoor bars and restaurants except in designated smoking areas. Since it allows designated smoking rooms, the tobacco control legislation in Vietnam does not follow the best practices as outlined by the FCTC Guidelines [7], which requires that designated smoking rooms should not be permitted for effective measures [7]. In addition, along with implementing the smoking ban regulations, restaurant owners have to deny any advertising activities for tobacco products in their restaurants. The staff have the right to request smokers to stop smoking in their restaurants and give fines for violations in accordance with the regulations. The Ministry of Health, Ministry of Public Security and Ministry of Industry and Trade have the responsibility to penalize restaurants that violate the regulations. The Communal People’s Committee takes prime responsibility to inspect the implementation of penalty towards smoking violations in public places that are under their management [29,30].

To date, evidence about the compliance with the tobacco control policy in Vietnamese restaurants is limited. A recent figure in GATS 2015 still showed a high prevalence of secondhand smoke (SHS) among restaurant customers (80.7%) [3]. However, GATS 2015 did not inform the rate of active smoking in the restaurants, how restaurants comply with the Law as well as the perceptions and attitudes of customers regarding the Law. Therefore, this study explored customers’ perceptions and attitudes towards the compliance of restaurants with the anti-smoking regulations, whether customers smoked or exposed to secondhand smoke in the restaurants. We also identified factors associated with smoking in restaurants. An evaluation based on the customer’s perspective gives a better understanding of how the laws have been complied with and suggests future actions for the Government to take to reinforce the laws [2,3,5].

## 2. Materials and Methods

### 2.1. Study Setting, Sample Size, and Sampling Method

We conducted a cross-sectional study in October 2015 in Hanoi—the capital of Vietnam. Hanoi has an area of over 3300 km^2^, including 30 districts (containing 581 communes) and a population of around 7 million. The population structure in Hanoi is mostly young people and the demand for utilizing food services is high. Hanoi is a particularly important economic and political center in Vietnam and has one of the largest foodservice industries in the country [31].

In this study, we prepared a list of 581 communes from 29/30 districts in Hanoi and used computer software to randomly select 176 communes. In each commune, based on the list of food facilities that had registered their business to local governmental authorities, the research team randomly chose 10 food facilities, resulting in 1760 food facilities in 176 communes. The convenient sampling method was applied to actively recruit the participants. In each selected facility, participants meeting the following inclusion criteria were invited to be involved in the study: (1) present at food establishments during the study period; (2) agreeing to participate and (3) having the capacity to answer the questionnaire. A total of 1760 customers were approached to give an interview (one customer per restaurant), however, data from only 1746 participants were eligible for analysis (99.2%) because participants did not answer the questionnaire completely.

### 2.2. Measures and Instruments

We carried out a pilot survey among fifty participants of different ages, genders, and occupations in the restaurants to examine the acceptability and the reliability of the questionnaire. Only minor changes to the wording were made to be appropriate for a participant’s preferences and culture. Face-to-face interviews were performed by ten students in the Master of Public Health Program and experts in the tobacco control field at Hanoi Medical University. The duration of each interview was 15–20 min.

Initially, the interviewers introduced briefly the purpose of the study, time for participation, and privacy procedures to potential participants; and asked them to provide written or verbal informed consent if they accepted to enroll. Then, we invited them to a convenient place in the restaurant to ensure their confidentiality and comfort. In each interview, a sufficient time was allowed for interviewees to adequately recall information. Participants did not receive any incentives for taking part in the survey.

In this study, before asking the questionnaire, we explained the definition of “restaurants” as: “A restaurant is defined as a place where meals are prepared and served to customers. Restaurants must have a specific menu for guests, arrangement of tables and chairs for serving purposes, dishes, food, drinks, etc. Restaurants also have equipment and food processing (kitchen)”. We obtained data of the following variables:

Socio-demographics: The socio-demographic characteristics included gender, marital status, education attainment, occupation, and age. Participants were also asked about kinds of food facilities that they often selected (including fast food restaurants, dine-in restaurants, and street food restaurants).

Smoking related-characteristics: Respondents were defined as a “current smoker” if they have smoked in the last 30 days and smoked at least 100 cigarettes in their life [32]. In this study, we estimated the 30-day prevalence of cigarette smoking in the restaurants among customers in order to avoid recall bias. Customers were classified as “restaurant smokers” if they were a current smoker and had smoked in restaurants within the last 30 days and as “restaurant non-smokers” if they had never smoked in a restaurant or if they (ever) had smoked in a restaurant but more than 30 days ago. We also investigated the experience of customers regarding secondhand smoke (SHS) in any restaurants in the previous 30 days. “Being exposed to tobacco smoke in restaurants” was used to define customers who passively smoked in restaurants in the last 30 days. Customers’ perceptions, attitudes, and complaints when they suffered from SHS were also investigated, including the frequency of exposure to SHS and whether they avoided restaurants because of tobacco smokers.

Prevention and Control of Tobacco Harms Law: The questions were based on the Law of Prevention and Control of Tobacco Harms [29]. We assessed participants’ knowledge on the Law and regulations regarding smoking bans in restaurants: “Have you ever heard or known about the Prevention and Control of Tobacco Harms Law?” and “Have you ever heard or known of regulations on prohibited smoking in restaurants except in designated smoking areas?”.

The study also examined the customers’ evaluations on the compliance with tobacco control policies among restaurants that they visited in the last 30 days. Respondents were asked about their perceptions of how restaurants had complied with the smoking control law using a 5-point Likert scale (All/Always; Almost/Usually; Mainly/Sometimes; Some/Rarely; None/Never).

(1) What is your overall assessment of the percentage of restaurants having no-smoking signs and designated smoking areas?
(2) If smoking in the restaurant in areas other than designated smoking areas, how often are smokers asked to stop smoking by staff?
(3) How often have you seen cigarette marketing in restaurants?

We also determined the attitudes of customers towards supporting the establishment of designated smoking areas as well as the sale of tobacco products in the restaurants.

### 2.3. Statistical Analysis

We analyzed data by using STATA software version 12.0 (StataCorp. LP, College Station, TX, USA). In this study, we applied a listwise deletion strategy (or complete case analysis) to handle the missing data [33]. *t*-test and χ^2^ test were employed to detect the differences in behaviors and attitudes related to tobacco control policies and SHS between smokers and non-smokers in restaurants.

In this study, we considered that customers (level 1 data) were clustered within communes (level 2 data), which were favorable to use a multilevel mixed effects logistic regression model. The multilevel model adjusted both levels as fixed effects and assumed the heterogeneity between communes [34]. We also assumed that the commune-level random effect of the intercept had a normal distribution with a mean of zero. Customer-related variables consisted of socio-demographic characteristics, use of restaurants (frequency and preferred types), and knowledge about the smoking ban in restaurants. Meanwhile, the location (urban/rural) was treated as a commune level predictor. A random intercept of the model was estimated by using commune identification (ID). Variance Inflation Factors (VIFs) were calculated to test the multicollinearity among variables, and a threshold value of 2.5 was used to identify multicollinearity [35]. A *p*-value < 0.05 was used to determine statistical significance.

### 2.4. Ethics Approval

The Institutional and Review Board (IRB) of the Hanoi Branch of Food Safety at the Hanoi Health Department authorized the study (Code 06/CCATVSTPHN). All participants received written informed consent after a clear explanation. Respondents were allowed to withdraw from the interview at any time, and this would not affect their usage of the food facilities. All personal information was kept confidential and anonymous data were only utilized for the research.

## 3. Results

A total of 1746 customers participated in this study and 38% of them were male. Nearly two–thirds were living with a spouse/partner and used food services. The percentage of participants who were office workers was 30.5%. Half of the respondents had above high school education (55.9%). The mean age was around 34.6 years (SD = 12.9). About 13.3% of customers were current smokers. Approximately 12% of respondents answered that they have smoked in restaurants they have visited (Table 1).

Table 2 demonstrates that the majority of customers were aware of the Law on Prevention and Control of Tobacco Harms (79%) as well as regulations on smoking bans in restaurants (78.4%). Only 10% reported that restaurants had designated smoking areas for smokers. More than three-fourths highlighted that restaurants did not have no-smoking signs. More than 70% of respondents rarely/never witnessed smoking-customers being reminded of smoking bans if they were smoking in the restaurant. The proportion of smokers supporting tobacco/waterpipe sales in the restaurant was four times higher than among non-smokers (37.5% and 8.8% respectively, χ^2^ test, *p* < 0.01). Only one-fifth of restaurant non-smokers (17.5%) selected restaurants that allowed smoking compared with nearly two-thirds of restaurant smokers (63.2%) (*p* < 0.01).

Table 3 presents that among 1721 participants, two-thirds reported that they were exposed to tobacco smoke or SHS in indoor restaurants. There was a large proportion of respondents feeling uncomfortable or unable to withstand passively smoking (69.3%). In addition, 96.7% of customers knew about the adverse effects of SHS. However, only 10% of customers regularly reminded others not to smoke in indoor restaurants.

Table 4 shows the results of multivariable regression models. All variables in the models had VIFs under 2.5, meaning that no multicollinearity was found. Those who were female (OR = 0.02, 95% CI = 0.01–0.05) were less likely to report their smoking in the restaurant than their counterparts. By contrast, people who had higher age (OR = 1.03; 95% CI = 1.01–1.06), high school education (OR = 2.14, 95% CI = 1.07–4.26), were office workers (OR = 3.24, 95% CI = 1.33–7.92) or unemployed (OR = 4.45; 95% CI = 1.09–18.15) had a higher likelihood of reporting to be restaurant smokers than those having lower high education or students, respectively. In addition, a greater likelihood of reporting smoking in the restaurants was associated with customers who often selected dine-in restaurants (OR = 1.62, 95% CI = 1.04–2.55) compared to those not often selecting restaurants.

## 4. Discussion

This current study evaluated the compliance with the Tobacco Control Law of restaurants from the customers’ perspective. In Vietnam, the law on the “Prevention and Control of Tobacco Harms”, promulgated in 2012, is a key milestone that has obtained many remarkable achievements in reducing smoking and SHS in public places, particularly in the expansion and development of the “non-smoking environment” model across the country [28,29]. Evaluations from the customers viewpoint provide empirical evidence about the feasibility, acceptability, and effectiveness of regulations to policy makers, and how to improve the implementation of the law.

In this study, the percentage of participants having knowledge of- and supporting smoke-free legislations was high. These results are in line with a previous national survey, which indicated that 82.3% of respondents were aware of the legislation and 85% of restaurant owners adopted indoor smoke-free policies in their restaurants [2,36]. However, our research also highlights that the level of perceived compliance with smoking ban regulations by customers was low. This proportion was lower than in similar studies in Thailand and Malaysia (56.9% and 39.4% respectively) [10]. The difference may be explained by the fact that the tobacco control law was promulgated in Thailand (2002) and Malaysia (2004) significantly earlier than in Vietnam (2012) [10]. Likewise, the number of non-smokers that prefer 100% smoke-free restaurants was much higher than the number of smokers who prefer smoke-filled restaurants. This finding demonstrates the need to implement 100% smoke-free restaurant environments, which followed the best practices of the FCTC Guidelines [7]. In addition, 8.7% of customers reported seeing cigarette advertising in restaurants, which is higher than the survey in 2010 (3.7%) [2]. This difference might have resulted from the fact that our study was conducted only in Hanoi, an epicenter of catering services in Vietnam and where advertising services are very popular. Despite the tobacco control law and inter-sectoral monitoring teams that have been formed and take primary responsibility to inspect enforcement, compliance with the law remains low and violations still existed, particularly among smaller restaurants businesses. This phenomenon may stem from the fact that monitoring is a complex task that faces many obstacles, such as there being greater profits generated with tobacco advertising, a lack of awareness regarding violation among small food retailers, a lack of human resources for monitoring, and a lack of collaboration between fields in enforcing violation penalties [28,37].

Notably, only a small number of respondents commented that staff reminded customers not to smoke in indoor restaurants except designated smoking areas. In the literature, people have argued that not allowing smoking indoors may cause a loss of revenue and will have an adverse impact on their business by reducing the number of smokers visiting [36,38,39]. Nevertheless, many studies have suggested that smoke-free laws did not hurt restaurant profits or sales and, in some cases, may also slightly increase business income [18,40,41,42]. Moreover, previous studies revealed that restaurant staff still lacked the enforcement skills as well as the persuasion skills to make customers extinguish their cigarettes [36,43]. Thus, hospitality workers should be trained and educated further about the dangers of tobacco as well as in skills for dealing with smoking customers in restaurants. The findings show that the attitude of non-smokers in enforcing others to stop smoking was similar to the attitude of staff, and it further emphasizes the role of promulgating stringent regulations on smoke-free restaurants to minimize passive smoking in restaurants.

One of the most important effects of the smoke-free law is the reduction of adverse effects from exposure to smoke [44]. However, in our study, a high proportion of participants reported to being exposed to tobacco smoke in restaurants in the last 30 days. This finding is consistent with the previous report in Vietnam in 2015 (80.7%) [3] and higher than in Thailand and the Philippines (34% and 21.9% respectively) [4], where the prevalence of tobacco use was approximately similar to Vietnam (Vietnam—22.5%, Thailand—24%, Philippines—23.8%) [3,45,46]. Although the majority of customers reported that they felt uncomfortable with SHS, only a small proportion of respondents requested smokers to stop smoking. A study conducted in Indonesia by Kaufman indicated that customers did not take personal action to avoid passive smoking and lacked the persuasion skills to make smoker sex distinguish their cigarettes [11]. At the same time, they would be more comfortable in asking smokers to obey the tobacco control law in restaurants where anti-smoking law signs were present [11]. Our results also support the relationship between being exposed to smoking in the restaurants and the advertisement of tobacco as well as the lack of no-smoking signs that have been explored in some previous studies [47,48]. In terms of smoking bans, the literature suggests that in many countries, smoking ban legislation could have a substantial impact on diminishing smoking intention as well as in increasing the attempts of smoking cessation [49,50]. However, restaurants in Vietnam still disregard smoking bans, especially small restaurants, which may lead to difficulty in monitoring the implementation of tobacco control laws in these facilities [36].

Several implications can be drawn from this study. Firstly, restaurant owners should fully comply with the tobacco control law by, for example, having smoke-free signs and banning any direct advertisement of cigarettes in their restaurants. It is also necessary to put smoking ban signs in places where the customers can easily see them. There is no risk-free level of secondhand smoke exposure, even brief exposure can be harmful to health and engineered measures (including ventilation, air filtration or designated smoking areas) do not fully protect customers against exposure to tobacco smoke [51,52]. Therefore, the best practice towards tobacco control law in restaurants is implementing 100% smoke-free environments.

Second, it is important for the Government to strengthen the monitoring and evaluation systems on compliance, especially in restaurants. More human resources should be allocated for monitoring and inspection. Local agencies need to improve their responsibility in the inspection of activities regarding the smoke-free law that are under their management in different provinces. Moreover, policymakers should pay more attention to the fundamental reasons for violations of the law and explain to restaurant owners that smoke-free regulation does not affect restaurant revenues or profits [18,40]. Providing “Smoke-Free Restaurant Practice Guides” along with accompanying training to restaurant owners, managers, and service staff is also essential.

Finally, our study suggests that the restaurant employees must be clearly informed about the harmful effects of secondhand smoke, the smoke-free policy, and their responsibilities under the ordinances to urge staff to fully comply with the law. Likewise, communication measures to raise awareness to smokers in terms of complying with the tobacco control law are also recommended.

Several limitations of this study should be acknowledged. First, the causal relationship between perceptions of smoking in restaurants and related factors cannot be established due to the cross-sectional design. We could only observe the prevalence of restaurants that did not comply with smoke-free regulation at one point in time. Second, data was collected by self-reports, which may lead to recall bias or socially desirable responding. Moreover, information about the degree of tobacco addiction was not included in the data collection process, requiring other studies to examine the effect of this factor to the compliance of the law. Finally, since the study only recruited participants in Hanoi, the sample was not representative of the general population, limiting the study in its ability to generalize about all of Vietnam. Thus, further studies should be conducted in order to help policymakers to have more specific ideas on how to reinforce smoke-free legislation.

## 5. Conclusions

Participants reported a high prevalence of restaurants that did not comply with the “Prevention and Control of Tobacco Harms” law, including a low percentage of restaurants that had no-smoking signs or where the advertisement of tobacco products still existed. Restaurant employees were reluctant to prompt customers to obey the tobacco control regulations. Customers experienced a high frequency of exposure to secondhand smoke in restaurants that they had visited in the last 30 days. The findings suggest that restaurant owners need to strictly comply with the tobacco control law, implementing 100% smoke-free environments as the best practice towards the law as well as improve their own inspections of their staff compliance with the law. In addition, our findings also emphasized that other relevant bodies should strengthen the magnitude and frequency of monitoring and inspecting compliance with smoke-free legislation among restaurants.

## Figures and Tables

**Table 1:** Demographic characteristics of respondents in the restaurants used.

Characteristics *	*N*	%
Gender (*n* = 1740)		
Male	663	38.1
Female	1077	61.9
Marital status (*n* = 1742)		
Single	592	34.0
Live with spouse/part	1120	64.3
Divorced/widow	30	1.7
Education attainment (*n* = 1724)		
<High school	224	13.0
High school	536	31.1
>High school	964	55.9
Occupation (*n* = 1743)		
Student	309	17.7
Unskilled worker	305	17.5
Office worker	531	30.5
Retired	130	7.5
Housewife	234	13.4
Unemployed	36	2.1
Others	198	11.4
Kind of food facilities often selected		
Fast food restaurant	715	41.2
Dine-in Restaurant	732	42.2
Street food restaurant	768	44.1
Current smokers	226	13.3
Have smoked in restaurants visited in the last 30 days (*n* = 1746)	207	11.9
	Mean	SD
Age (*n* = 1723)	34.6	12.9

* Some data were missing due to no response from participants.

**Table 2:** Customers’ perception of the compliance with “Prevention and control of tobacco harms law” according to smoke-free law of the restaurant.

Characteristics *	Restaurant Non-Smokers		Restaurant Smokers		Total		*p*-Value ^†^
	N	%	N	%	N	%	
Have heard of law on prevention and control of tobacco harms (*n* = 1669)	1163	78.5	157	84	1320	79.1	0.08
Have heard of regulations on the prevention of smoking in restaurants (*n* = 1450)	1005	79.6	132	70.2	1137	78.4	0.03
Percentage of restaurants having no-smoking signs (*n* = 1696)							
None	527	35.4	91	44.2	618	36.5	0.02
Rarely	597	40.1	70	34.0	667	39.3	
Some	211	14.1	35	17.0	246	14.5	
Almost all	118	7.9	8	3.8	126	7.4	
All	37	2.5	2	1.0	39	2.3	
Percentage of restaurants having designated smoking areas (*n* = 1726)							
None	992	65.3	153	74.2	1145	66.3	0.08
Rarely	389	25.6	42	20.4	431	25.0	
Some	49	3.2	6	2.9	55	3.2	
Almost all	29	1.9	1	0.5	30	1.7	
All	61	4.0	4	1.9	65	3.8	
Restaurants should have designated smoking areas (*n* = 1644)	1164	79.8	148	80	1312	79.8	0.94
Customers witness staff reminding smoking customers in restaurants (*n* = 1086)							
Never	386	43.8	66	32.4	452	41.5	0.04
Rarely	247	28.0	74	36.3	321	29.6	
Sometimes	167	18.9	41	20.1	208	19.2	
Usually	57	6.5	17	8.3	74	6.8	
Always	25	2.8	6	2.9	31	2.9	
Saw direct marketing to the users of tobacco (*n* = 1735)							
Never	726	47.5	87	42.4	813	46.9	<0.01
Rarely	393	25.7	48	23.4	441	25.4	
Sometimes	297	19.4	33	16.0	330	19.0	
Usually	95	6.2	14	6.8	109	6.3	
Always	19	1.2	23	11.2	42	2.4	
Restaurants should sell tobacco/waterpipes (*n* = 1691)	132	8.8	69	37.5	201	11.9	<0.01
Selecting restaurants that allow smoking (*n* = 1691)							
Yes	261	17.5	127	63.2	388	23	<0.01
No	370	24.8	12	6	382	22.6	
Depend on other conditions	859	57.7	62	30.9	921	54.5	

Statistical significance level *p* < 0.05; ^†^ Chi—square test; * Some data were missing due to no response from participants.

**Table 3:** Experiences of respondents regarding secondhand smoking in restaurants.

Characteristics *	*N*	%
Perceived frequency of exposure to tobacco smoke in a restaurant (*n* = 1721)		
Never	156	9.1
Rarely	430	25.0
Sometimes	811	47.1
Usually	248	14.4
Always	76	4.4
Discomfort when being exposed to tobacco smoke in a restaurant (*n* = 1735)		
Not discomfort	206	11.9
Somewhat	328	18.9
Uncomfortable	596	34.4
Very uncomfortable	459	26.5
Unable to withstand	146	8.4
Complaining to smokers in the restaurant (*n* = 1735)		
Never	659	38.0
Rarely	524	30.2
Sometimes	371	21.4
Usually	118	6.8
Always	63	3.6

* Some data were missing due to no response from participants.

**Table 4:** Factors associated with smoking in the restaurant (*n* = 1352).

Characteristics	Restaurant Smokers	
	OR	95% CI
Gender (Female vs. Male)	0.02 ***	0.01; 0.05
Age	1.03 ***	1.01; 1.06
Education Level attained (vs. <High school)		
High school	2.14 **	1.07; 4.26
>High school	0.95	0.44; 2.03
Occupation (vs. Student)		
Unskilled worker	2.07	0.80; 5.36
Office worker	3.24 **	1.33; 7.92
Retirement	1.55	0.44; 5.40
Housemaker	1.73	0.45; 6.56
Unemployed	4.45 **	1.09; 18.15
Others	4.03 ***	1.55; 10.46
Location (Urban vs. Rural)	1.02	0.54; 1.94
Kind of food facilities often selected		
Dine-in Restaurant (Yes vs. No)	1.62 **	1.04; 2.55
Street food restaurants (Yes vs. No)	0.67 *	0.43; 1.05
Have heard of tobacco harm control Law (Yes vs. No)	0.74	0.35; 1.57
Have heard of regulations about smoking bans in restaurants (Yes vs. No)	0.71	0.43; 1.20

* Indicate significance level *** *p* < 0.01, ** *p* < 0.05 by multivariate logistic regression. OR: Odds Ratio; 95% CI: 95% Confidence Interval.

## Abbreviations

WHO	World Health Organization
WHO FCTC	WHO Framework Convention on Tobacco Control
SHS	Secondhand smoke
GATS	Global Adult Tobacco Survey
IRB	Institutional and Review Board
